# Outcomes and toxicity of concomitant radioimmunotherapy following PD-1 blockade for locally advanced and metastatic cutaneous squamous cell carcinoma

**DOI:** 10.3389/fonc.2025.1679699

**Published:** 2025-11-20

**Authors:** Ari A. Kassardjian, Colton J. Ladbury, Andrew Tam, Sean Maroongroge, Yan Xing, Ramya Muddasani, Badri Modi, Arya Amini

**Affiliations:** 1 Department of Radiation Oncology, City of Hope, Duarte, CA, United States; 2 Department of Medical Oncology, City of Hope, Duarte, CA, United States; 3 Department of Dermatology, City of Hope, Duarte, CA, United States

**Keywords:** cutaneous squamous cell carcinoma (cSCC), immune checkpoint inhibitor (ICI), stereotactic body radiation therapy (SBRT), intensity modulated radiation therapy (IMRT), immunotherapy, concurrent radiation therapy

## Abstract

**Introduction:**

Immune checkpoint inhibitors (ICI) effectively treat advanced cutaneous squamous cell carcinoma (cSCC), yet some patients continue to have disease progression. Combining radiation therapy (RT) with ICI represents a potential therapeutic option, yet limited data exist regarding oncologic outcomes and safety profile.

**Methods:**

This retrospective cohort study examined patients treated with concurrent ICI and RT between April 2019 and November 2022 and stratified by locally advanced or metastatic status. Outcomes included locoregional control (LRC), freedom from distant metastases (FFDM), progression-free survival (PFS), overall survival (OS), and toxicity. Statistical analysis was performed using Kaplan-Meier or Fine-Gray competing risk survival analyses.

**Results:**

Thirteen patients (median age 77 years) with locally advanced (53.8%) or metastatic (46.2%) cSCC on cemiplimab (84.6%) or pembrolizumab (15.4%) received concomitant RT using intensity-modulated radiotherapy (69.2%) or stereotactic body radiotherapy (30.8%). With median follow-up of 15.4 months, overall 1-year and 2-year outcomes were OS: 75.2% and 62.7%; PFS: 59.8% and 25.6%; FFDM: 83.8% and 62.4%; LRC 100% and 84.3%, respectively. Locally advanced patients had significantly greater LRC than metastatic patients (100% *vs*. 56.3%; p<0.001), but no significant difference in PFS, FFDM, or OS. Only one patient experienced grade 3 radiation dermatitis, with no grade 4+ toxicities.

**Conclusion:**

Radioimmunotherapy demonstrated favorable oncologic outcomes with minimal toxicity. Addition of consolidative RT to ICI therapy may represent a safe and effective approach for this challenging patient population, warranting further prospective investigation.

## Introduction

With an estimated incidence of 1.8 million cases per year, cutaneous squamous cell carcinoma (cSCC) afflicts significant morbidity on the US population (1). The National Comprehensive Cancer Center (NCCN) stratifies mortality risk of a primary cSCC lesion into low, high, or very high-risk based on clinicopathologic characteristics including size, location, depth of invasion, perineural invasion (PNI), and others (2). An estimated 4000–8000 patients die of cSCC annually, and the majority of these deaths arise from patients with high-risk or very high-risk disease (3). Historically, cSCC patients with local lymph node involvement exhibit 2-year overall survival (OS) of 69% to 83%, and increasing nodal burden correlates with poorer outcomes (4). Patients with distant metastases, arising in 2% of cSCC patients, harbor a range of survival outcomes at 2-years of 38% to 64%, whereby mortality may be 2-fold higher in immunocompromised patients (4–6).

When possible, surgical resection to clear margins remains the gold standard treatment for cSCC (7). Radiation therapy (RT) is typically reserved for primary treatment of unresectable tumors, or RT can be delivered adjuvantly following surgery to reduce risk of recurrence in tumors with high-risk features, such as PNI, positive margins, tumor stage of T3 or higher, parotid gland involvement, or in the setting of immunosuppression (7–9). Furthermore, adjuvant radiation may improve survival in tumors with extensive PNI, lymph node positivity, or metastatic disease (10).

Since 2018, immune checkpoint inhibitors (ICI) have demonstrated significant activity in the treatment of locally advanced and metastatic cSCC no longer amenable to curative local therapy (11). Both cemiplimab and pembrolizumab are approved by the United States Federal Drug Administration (FDA) in advanced cSCC, and there is now evidence that ICI therapy may benefit patients if used neoadjuvantly for earlier stage, resectable disease (12, 13).

While ICI treatment of advanced cSCC can result in dramatic improvement, approximately 50% of patients with advanced cSCC do not respond to ICI (14, 15). These patients may still benefit from RT, yet few series have thus far reported on the outcomes of concurrent use of RT and ICI therapy in locally advanced and metastatic cSCC, and their combined use is not well understood (16). Given the concern of additive toxicities among these two modalities, we set out to review 13 cases of advanced cSCC with clinical progression on ICI monotherapy, to which RT was added to enhance disease control while maintaining an overall acceptable toxicity profile. In this study, we examine a cohort of patients with cSCC treated with RT and ICI and report rates of oncologic control, survival, and toxicity.

## Materials and methods

### Study design

We examined a retrospective cohort of patients at a single institution who received concurrent ICI and RT between April 2019 and November 2022. All patients were at least 18 years of age with tissue diagnosis confirming cSCC. RT was delivered using either intensity modulated radiotherapy (IMRT) or stereotactic body radiotherapy (SBRT) techniques. Concurrent radioimmunotherapy was defined as ICI infusion within three weeks before or after RT delivery. Deidentified patient data was collected from electronic medical records (EMR), and the study was approved by the Institutional Review Board and conducted following the ethical tenets of the Helsinski Declaration.

### Data collection

The EMR provided information regarding patient demographic, clinical, oncologic, and treatment data. Subjects were characterized as having locally advanced or metastatic cSCC based on disease extent during initial staging work-up and multidisciplinary tumor board discussion. Tumor (T) stage was determined using the American Joint Committee on Cancer (AJCC) *Cancer Staging Manual*, 8^th^ edition. Nodal (N) stage for patients with cSCC of the head and neck (H&N) was defined based on non-HPV-associated H&N cancers. For cSCC arising from the body, patients were deemed either node-positive (N1) or node-negative (N0). Patients were considered metastatic (M1) by distant spread to organs or non-regional lymph nodes. Toxicity data was collected at each visit using the Common Terminology Criteria for Adverse Events version 5.0 (CTCAE v5.0). Tabulated toxicity data incorporated the highest grade acute and late toxicity documented in the EMR for each specific patient.

### Study endpoints and statistical analysis

Patients were monitored with clinical examination and imaging, either with computed tomography (CT), positron emission tomography (PET), and/or magnetic resonance imaging (MRI), performed at minimum every 12 weeks, or as clinically indicated. Radiographic response was assessed using the Response Evaluation Criteria in Solid Tumors (RECIST) v1.1 criteria for patients with measurable cSCC.

Endpoints were analyzed at 1 year and 2 years following completion of RT. Locoregional control (LRC) was defined as the absence of disease progression or recurrence in the radiation field, first echelon draining lymph node basin, or in-transit tissue. Similarly, freedom from distant metastases (FFDM) was attributed to distant progression, either from new or enlarging metastatic tumor deposits noted on physical examination or imaging. Progression-free survival (PFS) was defined as any disease progression, or any death event. Patients without progression were censored at last follow-up. Finally, OS was calculated using death from any cause after completion of RT. Statistical analyses and comparison between groups were performed using Kaplan-Meier or Fine-Gray competing risk survival analyses, as appropriate. 95% confidence interval (CI) is also reported for each endpoint. The date of last follow-up was January 30, 2023. Given the small sample size, the statistical comparisons between groups should be considered exploratory in nature, and p-values interpreted with appropriate caution.

## Results

Thirteen patients with either locally advanced (53.8%) or metastatic (46.2%) cSCC received concurrent RT while receiving cemiplimab (84.6%) or pembrolizumab (15.4%) (**Table 1**). Median age was 77 years (range 65-92), and all patients were male. Most patients (84.6%) were non-Hispanic White. Median Karnofsky performance status (KPS) was 80 (range 70-90). All identified primary cSCC lesions were at least T3, and all patients had very high-risk disease based on the NCCN cSCC guidelines (2). Most patients (61.5%) had lymph node involvement or distant metastases, and almost half of the cSCC lesions (46.2%) were T4a or T4b (**Table 2**). Radiation dose and fractionation ranged from 55 to 70 Gray (Gy) in 20 to 35 daily fractions for IMRT (69.2%), and 30 to 40 Gy for SBRT (30.8%) in 5 fractions delivered every other day.

**Table 1:** Demographic characteristics of overall cohort of patients receiving radiation and immune checkpoint inhibitor therapy.

Variable	Patients (n=13)
Gender	
Male	13 (100%)
Median Age (SD, Range)	76.9 years (9.1, 65-92 years)
Race and ethnicity	
Non-Hispanic White	11 (84.6%)
Hispanic White	1 (7.7%)
Asian	1 (7.7%)
KPS, Median (Range)	80 (70-90)
Disease type	
Locally advanced	7 (53.8%)
Metastatic	6 (46.2%)
Irradiated site	
Head and Neck	8 (61.5%)
Body	5 (38.4%)
Radiation technique, dose range (Gy)	
IMRT	9 (69.2%, 55-70)
SBRT	4 (30.7%, 30-40)
PD-1 inhibitor	
Cemiplimab	11 (84.6%)
Pembrolizumab	2 (15.4%)

KPS, Karnofsky Performance Status; IMRT, Intensity Modulated Radiation Therapy; SBRT, Stereotactic Body Radiation Therapy; PD-1, Programmed Cell Death Protein 1; Gy, Gray; SD, Standard Deviation.

**Table 2:** Patient and tumor characteristics.

Patient #	Sex	Age	KPS	Race/Ethnicity	T stage	N stage	M stage	AJCC group stage	Site	Site details	Radiation technique	Dose (Gy)/ #fractions	PD-1 inhibitor	Acute toxicities	Late Toxicities	Event	Disease status
1	M	65	90	Hispanic White	T3	N1	M1	Stage IVC	Body	Foot	SBRT	30/5	Cemiplimab	Grade 1 nausea	N/A	LRR	Expired
2	M	92	70	Non-Hispanic White	T3	N1	M1	Stage IVC	Body	Axilla	SBRT	35/5	Pembrolizumab	Grade 2 dermatitis	N/A	Death	Expired
3	M	87	70	Non-Hispanic White	T3	N0	M0	Stage III	H&N	Scalp, ear, and parotid	IMRT	56/28	Cemiplimab	Grade 2 dermatitis and pain	N/A	Death	Expired
4	M	71	70	Non-Hispanic White	T4b	N0	M0	Stage IVB	H&N	Cheek, orbit, and base of skull	IMRT	55/20	Pembrolizumab	Grade 2 mucositis	Grade 1 dry mouth, fibrosis	None	Stable
5	M	77	80	Non-Hispanic White	T4a	N1	M1	Stage IVC	Body	Arm	IMRT	55/20	Cemiplimab	Grade 3 dermatitis	Grade 1 fibrosis, telangiectasia	DM	Lung mets
6	M	72	70	Non-Hispanic White	T4b	N0	M0	Stage IVB	H&N	Scalp and skull/dura mater	IMRT	70/35	Cemiplimab	Grade 2 fatigue, grade 1 dermatitis	Grade 2 alopecia	DM	Brain mets
7	M	81	80	Asian	T4b	N0	M0	Stage IVB	H&N	Scalp and skull/dura mater	IMRT	70/35	Cemiplimab	Grade 1 pain and pruritus	N/A	None	Stable
8	M	70	80	Non-Hispanic White	T3	N2b	M0	Stage IVA	H&N	Face, parotid, and neck	SBRT	40/5	Cemiplimab	Grade 1 xerostomia	Grade 1 fibrosis	None	Stable
9	M	89	80	Non-Hispanic White	TX	N1	M1	Stage IVC	Body	Axilla	SBRT	35/5	Cemiplimab	N/A	N/A	DM	Expired
10	M	77	70	Non-Hispanic White	T4b	N0	M1	Stage IVC	H&N	Scalp and cheek	IMRT	55/20	Cemiplimab	N/A	N/A	DM	Mediastinal progression
11	M	65	90	Non-Hispanic White	T3	N2b	M0	Stage IVA	H&N	Temple, parotid, and neck	IMRT	66/33	Cemiplimab	Grade 2 dermatitis, esophagitis	Grade 1 fibrosis, trismus	None	Stable
12	M	87	90	Non-Hispanic White	T4b	N0	M0	Stage IVB	H&N	Scalp and skull/dura mater	IMRT	70/35	Cemiplimab	Grade 2 dermatitis	N/A	Death	Expired
13	M	67	90	Non-Hispanic White	TX	N1	M1	Stage IVC	Body	Axilla	IMRT	66/33	Cemiplimab	N/A	N/A	None	Stable

M, Male; KPS, Karnofsky Performance Status; H&N, Head and Neck; IMRT, Intensity Modulated Radiation Therapy; SBRT, Stereotactic Body Radiation Therapy; Gy, Gray; LRR, Locoregional Recurrence; DM, Distant Metastasis; PD-1, Programmed Cell Death Protein 1.

Most patients underwent RT to the H&N (61.5%) Three patients had parotid gland involvement, which was included in the radiation field. Of those, two had N2b disease with multiple pathologic ipsilateral neck lymph nodes, while the third was N0. In the other five H&N cSCC patients, all had T4b lesions with skull bone invasion, and none had positive neck lymph nodes. Seven out of eight total H&N patients received IMRT while the eighth received SBRT. One patient who received H&N RT had M1 disease with mediastinal lymphadenopathy.

Regarding the patients receiving RT to the body (38.5%), four underwent radiation to a proximal upper extremity and/or axilla while one had treatment of a lower extremity. All five patients who received RT to the body had node-positive and metastatic disease. Two patients who underwent RT to axillary lesions had unknown primary cutaneous sites and were designated TX. Three patients underwent SBRT while the other two received IMRT.

**Figure 1:**
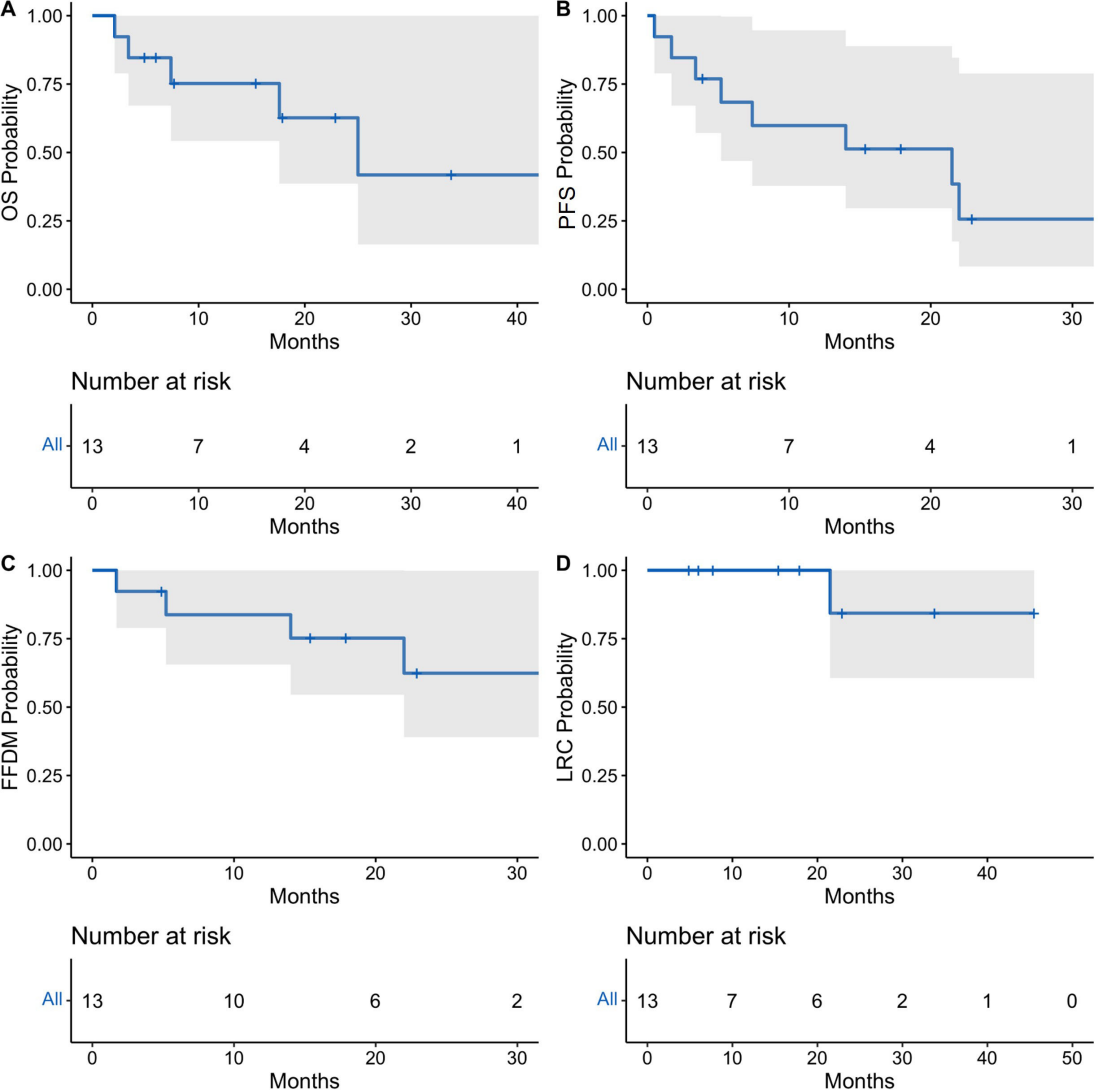
Oncologic outcomes for total cohort of patients receiving concurrent radioimmunotherapy. Kaplan-Meier estimates showing probability of overall survival **(A)**, progression-free survival **(B)**, freedom from distant metastasis **(C)**, and locoregional control **(D)**. Tick-marks indicate censored data. Data was analyzed at 12 and 24 months. 95% confidence intervals denoted by shaded areas.

Median follow-up was 15.4 months overall and 16.7 months for living patients. At final follow-up, eight patients remained alive, and five were without disease progression following radioimmunotherapy. Outcomes were analyzed at 1 and 2 years (**Figure 1**). Median OS was 25 months [95% CI: 17.6 months to not reached (NR)], and 1-year and 2-year median OS were 75.2% [95% CI: 54.2%-100%] and 62.7% [95% CI: 38.6%-100%], respectively (**Table 3**). Median PFS was 21.5 months [5.2 months to NR], and 1-year median PFS was 59.8% [95% CI: 37.8%-94.7%] while 2-year median PFS decreased to 25.6% [95% CI: 8.3%-78.9%]. There was also a drop in median FFDM from 83.8% [95% CI: 65.6%-100%] at 1 year compared to 62.4% [95% CI: 39.0%-99.8%] at 2 years. Finally, median LRC among the entire patient cohort was 100% [95% CI: 100%-100%] at 1 year and 84.3% [95% CI: 60.6%-100%] at 2 years. In-field local control was 100% in both groups at 1-year and 2-years, and there were no in-field local failures following RT.

**Table 3:** Summary of oncologic outcomes after concurrent radioimmunotherapy. There was a statistically significant difference of locoregional control between locally advanced and metastatic cohorts (p<0.001).

Category	Locally advanced cohort (n=7)	Metastatic cohort (n=6)	Total (N = 13)	*P* value
Overall survival				
1-year, Median % (95% CI)	68.6 (40.3-100)	83.3 (58.3-100)	75.2 (54.2-100)	0.4
2-year, Median % (95% CI)	68.6 (40.3-100)	41.7 (10-100)	62.7 (38.6-100)	
Progression-free survival				
1-year, Median % (95% CI)	68.6 (40.3-100)	50.0 (22.5-100)	59.8 (37.8-94.7)	0.11
2-year, Median % (95% CI)	45.7 (17.5-100)	0 (0-0)	25.6 (8.3-78.9)	
Freedom from distant metastasis				
1-year, Median % (95% CI)	100 (100-100)	66.3 (37.8-100)	83.8 (65.6-100)	0.09
2-year, Median % (95% CI)	76.6 (45.7-100)	49.3 (22.3-100)	62.4 (39.0-99.8)	
Locoregional control				
1-year, Median % (95% CI)	100 (100-100)	100 (100-100)	100 (100-100)	<0.001
2-year, Median % (95% CI)	100 (100-100)	56.3 (19.6-100)	84.3 (60.6-100)	

There was no difference among the other categories.

CI, Confidence Interval.

**Figure 2:**
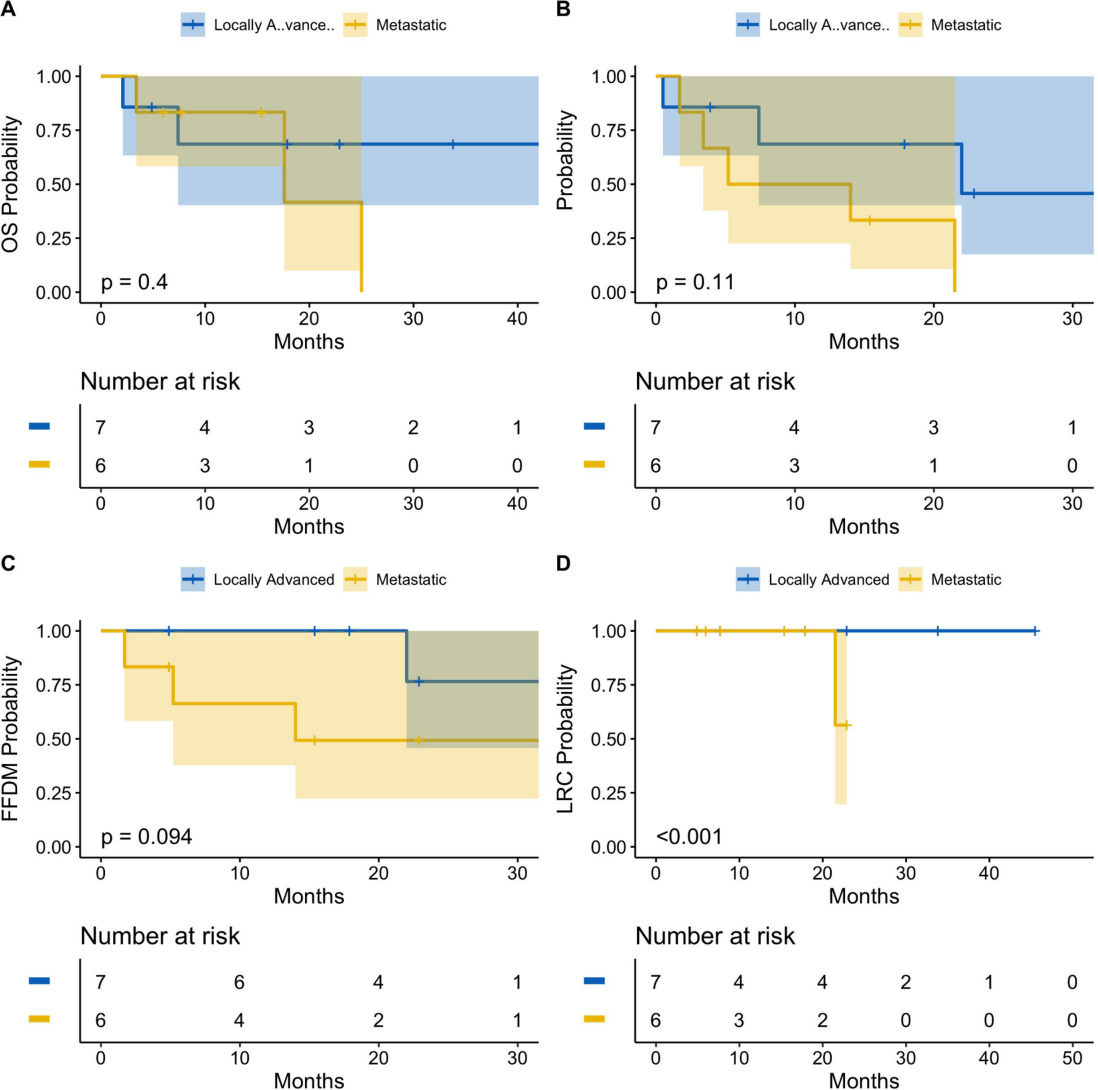
Oncologic outcomes following radioimmunotherapy stratified by locally advanced or metastatic disease status at baseline. Kaplan-Meier estimates showing probability of overall survival **(A)**, progression-free survival **(B)**, freedom from distant metastasis **(C)**, and locoregional control **(D)**. There was a statitstically significant difference of locoregional control between locally advanced and metastatic patients (p<0.001). There was no significant difference between the two cohorts for overall survival, progression-free survival, or freedom from distant metastasis. Tick-marks indicate censored data. Data analyzed at 12 and 24 months. 95% confidence intervals denoted by shaded areas. Blue: Locally advanced. Gold: Metastatic.

Patients were also stratified by locally advanced or metastatic status at the time of radioimmunotherapy (**Figure 2**). Both locally advanced and metastatic cSCC patients had a median probability of LRC of 100% [95% CI: 100%-100%] at 1 year. At 2 years, median LRC remained 100% [95% CI: 100%-100%] for locally advanced patients, while median LRC for metastatic patients decreased to 56.3% [95% CI: 19.6%-100%]. Patients with locally advanced cSCC had significantly greater LRC compared to patients with metastatic disease (p<0.001).

Median OS was 68.6% [95% CI: 40.3%-100%] for locally advanced patients at both 1-year and 2-years, versus 83.3% [95% CI: 58.3%-100%] and 41.7% [95% CI: 10.0%-100%] for metastatic patients at 1-year and 2-years, respectively. There was no statistically significant difference in median OS observed between cohorts (p=0.4). Similarly, patients with locally advanced cSCC had a median PFS probability of 68.6% [95% CI: 40.3%-100%] at 1-year and 45.7% [95% CI: 17.5%-100%] at 2-years, compared to patients with metastatic cSCC, who had a median 1-year PFS of 50.0% [95% CI: 22.5%-100%] and 2-year PFS of 0.0% [95% CI: 0%-0%]. Again, there was no statistically significant difference in PFS between locally advanced and metastatic patients (p=0.11). Finally, after 1 year, the median FFDM probability for locally advanced patients was 100% [95% CI: 100%-100%], which decreased to 76.6% [95% CI: 45.7%-100%] at 2-years, compared to 66.3% [95% CI: 37.8%-100%] and 49.3% [95% CI: 22.3%-100%] for metastatic patients at 1-year and 2-years, respectively. There was no statistically significant difference of FFDM between locally advanced and metastatic cSCC patients (p=0.094).

**Figure 3:**
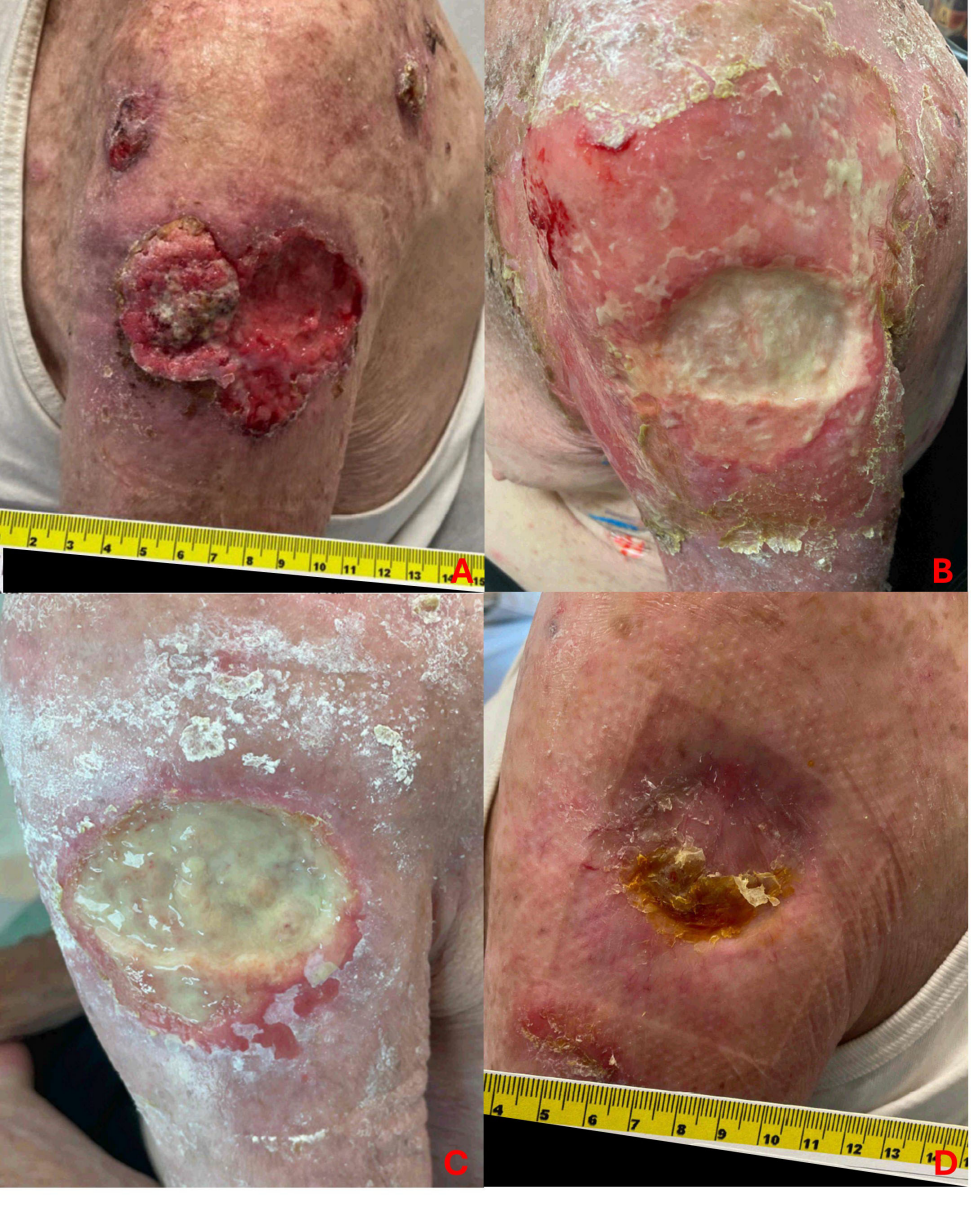
Acute grade 3 radiation dermatitis. One patient experienced acute grade 3 radiation dermatitis following IMRT of a ~7 cm ulcerative lesion with two additional satellite nodules located superiorly on the anterior and posterior left shoulder. Image panels show pre-radiation **(A)**, followed by moist desquamation and minor bleeding at 1-week post-radiation **(B)**. At 1-month **(C)**, there is evidence of granulation tissue and healing by secondary intention. With general wound care and antimicrobial dressing, his lesion healed with expected mild fibrosis and telangiectasia, seen at 8-months post-radiation **(D)**.

No patient experienced grade 4 or 5 radiation-related toxicities. The complete list of acute and late toxicities is tabulated in **Table 2**. One patient experienced acute grade 3 radiation dermatitis. Prior to radioimmunotherapy treatment, he presented with a 7 centimeter (cm) ulcerative lesion on his left proximal arm with two satellite nodules located superiorly on the anterior and posterior shoulder (**Figure 3**). He initially started cemiplimab for 6 months without significant response, and he subsequently underwent IMRT to a total dose of 55 Gy in 20 fractions to improve local control of his left arm ulcerative lesion. About 10 days after delivery of IMRT, he developed moist skin desquamation with superficial bleeding. He underwent regular wound care with medicated foam dressings to facilitate healing via secondary intention, and he did not require any opioids for pain control or negative pressure wound therapy. By his one-month follow-up, his satellite lesions fully regressed and the surrounding radiation dermatitis had also resolved. There was persistent ulceration of his main lesion with granulation tissue and overlying exudate. By his eight-month follow-up, there was complete healing via secondary intention with minor long-term toxicities from radiation, consisting of grade 1 fibrosis and telangiectasia.

## Discussion

Historically, the role of radiotherapy in locally advanced and metastatic cSCC is not well defined. Retrospective studies of definitive RT for cSCC have demonstrated that the degree of tumor control correlates with size and stage; T3 and T4 lesions have local control rates anywhere from 75% to 85%, and disease-specific survival at 3 years is about 38% (17, 18). With adjuvant RT, the strongest benefit has been seen in patients with PNI or regional metastatic disease, and addition of RT may improve survival in high-risk patients (10).

Our study examined tumor control, oncologic outcomes, and the toxicity profile of 13 patients receiving RT for either locally advanced or metastatic cSCC with progression on ICI monotherapy. Other than LRC, there were no statistically significant differences in outcomes between locally advanced and metastatic patients. Notably, there were no in-field local failures following RT, and one patient had a regional recurrence along the ankle two years following SBRT to his toe and inguinal lymph nodes. Thus, patients had excellent LRC overall following radioimmunotherapy, suggesting a possible benefit of RT in this population. Importantly, about 50% of patients on ICI monotherapy will fail to respond or have progressive disease (12–15). Under these circumstances, RT may provide a durable LRC benefit of locally aggressive cutaneous lesions causing significant morbidity without significantly increasing toxicity.

Median OS of the entire cohort was 25 months, similar to data from a single-institution study of definitive RT that showed a median OS of 19 months in inoperable stage III and IV cSCC patients (19). After combining operable and inoperable patients, the addition of a PD-1 inhibitor (n=20) yielded a 1-year OS of 84% [95% CI: 67.5-100%] and 2-year OS of 75.7% [54.1-96.9%], compared to OS observed in our cohort of 75.2% [54.2-100%] and 62.7% [38.6-100%] at 1-year and 2-years, respectively. However, the vast majority of patients in our study population had more aggressive tumors with PNI or metastatic disease, and in their study, OS of inoperable patients alone on ICI therapy was not reported.

With cemiplimab monotherapy, 1-year OS has been reported at 93%, and the estimated proportion of patients alive and without disease progression after 1 year is 58% [95% CI: 44-70%] among locally advanced cSCC patients without nodal or distant metastases (15). With the addition metastatic patients, the estimated probability of median OS at 1-year decreases to 81% [95% CI: 68%-89%], and 1-year PFS is 53% [95% CI: 37%-66%] (14). In our overall cohort, 1-year OS was 75.2% [95% CI: 54.1%-100%] and 1-year PFS was 59.8% [95% CI: 37.8%-94.7%]. Importantly, cSCC patients in our population were not well-controlled on ICI therapy, prompting consideration of RT, and progression events almost exclusively consisted of distant metastatic progression and death. RT may represent a feasible option not only to boost local control, but potentially also preserve the patient’s current line of ICI therapy without transitioning to a different systemic agent.

As ICI have become standard treatments for advanced cSCC, radiation oncologists are increasingly consulted to integrate RT for local control of patients with progressive or symptomatic disease. The combination of anti PD-1 agents and RT not only modifies the local tumor immune microenvironment, but it also may impact overall systemic disease control via the abscopal effect (20). By inducing double-stranded DNA breaks and immunogenic cell death, RT propagates release of cytokines and tumor antigens, which subsequently activate cytotoxic CD8+ T-cells (21). Increased cytosolic DNA activates the cyclic GMP-AMP synthase stimulator of interferon genes (cGAS-STING) protein pathway, further propagating a pro-inflammatory tumoricidal response in tumor-draining lymph nodes and regional lymphatics (22). Implementation of ICI sustains this antitumor activity and promotes long-term immune memory, and ultimately promotes both local and systemic disease control (23). Site selection and RT dose become critical, as lower doses may stimulate the immune system, while high dose per fraction to regional lymphatics may induce leukopenia and dampen systemic immunity (24).

Clinically, the observation of an abscopal effect (i.e., when RT induces distant tumor control) is elusive in practice. However, combined radioimmunotherapy with ICI and RT may boost LRC in locally advanced and metastatic cSCC without a significant increase in treatment-related toxicity (25, 26). Recent data from Israel demonstrated that RT and PD-1 inhibitors yields durable response rates in locally advanced and metastatic cSCC, with RT delivered before, during, or after ICI (27). Another study from Italy personalized dose and fractionation, employing conventionally fractionated RT over 6–7 weeks when feasible, or delivering a hypofractionated course over 1–2 weeks for older and/or frailer patients (25). Our series employed a similar individualized approach, tailoring dose and fractionation based on performance status, treatment site, and overall disease burden. Prospective trials are needed to further define optimal RT dose and sequencing in combination with ICI.

Overall, limited toxicities were observed in this study. There was one grade 3 acute toxicity following IMRT to a large, ulcerative lesion on the left shoulder with concurrent cemiplimab. Within 1 week following completion of RT, there was evidence of moist desquamation and superficial bleeding in areas other than skin folds, necessitating designation of acute grade 3 dermatitis. Otherwise, there were no other grade 3 or higher acute toxicities, no grade 3 or higher late toxicity, and no patients experienced any treatment-related break. One 87-year-old patient transitioned to hospice and stopped IMRT at 56 Gy instead of 70 Gy due to distant disease progression.

Several limitations exist in the present study. Similar to other retrospective studies of advanced cSCC, patients had an older median age of 77 years old, and almost all patients harbored T4, node-positive, and/or metastatic disease at baseline. Moreover, all patients in the study were male, most were non-Hispanic White, and many had significant medical comorbidities or immunocompromised status. Despite the limited patient number, this was a heterogeneous patient population with stage III or stage IV cSCC involving many different cutaneous H&N or body areas.

Five patients had undergone surgical resection with adverse pathologic features (i.e., positive margins or PNI) or local recurrence, prompting subsequent ICI therapy followed by additional local therapy with RT combined with ICI. Another 5 patients initiated treatment with ICI due to unresectable disease for at least 4–6 cycles, and RT was subsequently added for locoregional control. The remaining patients initiated combined radioimmunotherapy up-front. This heterogeneity, although a limitation of our analysis, represents a real-world application of this approach across different clinical contexts.

Furthermore, follow-up was limited in some instances, resulting in significant censoring of 2-year outcome estimates and larger 95% CI at that timepoint. These 2-year endpoints should be interpreted as exploratory, and additional multi-center prospective trials are needed to better define long-term response rates, oncologic outcomes, and safety profiles of combined RT and ICI therapy. There is a randomized, phase 3 study (NCT03969004) currently enrolling high-risk cSCC patients to receive either cemiplimab or placebo following surgery and adjuvant radiation, which will further evaluate outcomes and toxicity in a similar patient population (28).

## Conclusions

In this retrospective study of locally advanced and metastatic patients with cSCC not well-controlled on ICI therapy, the addition of concurrent RT was overall well tolerated, and oncologic outcomes were similar to published endpoints for this challenging patient population. Both acute and late toxicities were overall acceptable, and the addition of RT may provide a benefit to LRC, yet future prospective trials combining the two modalities are necessary to better define the role of RT in advanced cSCC as novel ICI therapies continue to emerge. Overall, our findings suggest there may be a combined benefit of radioimmunotherapy in advanced cSCC without the expense of increased toxicity.

## Data Availability

The raw data supporting the conclusions of this article will be made available by the authors, without undue reservation.
